# Importance of overstorey attributes for understorey litter production and nutrient cycling in European forests

**DOI:** 10.1186/s40663-020-00256-x

**Published:** 2020-07-12

**Authors:** Dries Landuyt, Evy Ampoorter, Cristina C. Bastias, Raquel Benavides, Sandra Müller, Michael Scherer-Lorenzen, Fernando Valladares, Safaa Wasof, Kris Verheyen

**Affiliations:** 1 Forest & Nature Lab, Department of Environment, Ghent University, Ghent, Belgium; 2 LINCGlobal, Department of Biogeography and Global Change, National Museum of Natural Science-CSIC, Madrid, Spain; 3 Faculty of Biology, Geobotany, University of Freiburg, Freiburg im Breisgau, Germany

**Keywords:** FunDivEUROPE, Nutrient cycling, Litter production, Understorey, Overstorey, Tree species richness, Light availability, Litter quality, Proportion evergreen tree species

## Abstract

**Background:**

In contrast with the negligible contribution of the forest understorey to the total aboveground phytobiomass of a forest, its share in annual litter production and nutrient cycling may be more important. Whether and how this functional role of the understorey differs across forest types and depends upon overstorey characteristics remains to be investigated.

**Methods:**

We sampled 209 plots of the FunDivEUROPE Exploratory Platform, a network of study plots covering local gradients of tree diversity spread over six contrasting forest types in Europe. To estimate the relative contribution of the understorey to carbon and nutrient cycling, we sampled non-lignified aboveground understorey biomass and overstorey leaf litterfall in all plots. Understorey samples were analysed for C, N and P concentrations, overstorey leaf litterfall for C and N concentrations. We additionally quantified a set of overstorey attributes, including species richness, proportion of evergreen species, light availability (representing crown density) and litter quality, and investigated whether they drive the understorey’s contribution to carbon and nutrient cycling.

**Results and conclusions:**

Overstorey litter production and nutrient stocks in litterfall clearly exceeded the contribution of the understorey for all forest types, and the share of the understorey was higher in forests at the extremes of the climatic gradient. In most of the investigated forest types, it was mainly light availability that determined the contribution of the understorey to yearly carbon and nutrient cycling. Overstorey species richness did not affect the contribution of the understorey to carbon and nutrient cycling in any of the investigated forest types.

## Background

Studies on forest ecosystem functioning generally focus on trees and their functional characteristics, while ignoring the understorey, the herbs, grasses, ferns, dwarf shrubs, mosses and seedlings growing at the forest floor. Despite their small stature, understorey plants can play an important functional role, especially in open forests where overstorey productivity is low (Landuyt et al. 2019a). In such forests, the understorey can provide up to 41% of a forest’s annual litterfall (Gilliam 2007; Muller 2014; Landuyt et al. 2019a). Moreover, as leaf-level nutrient concentrations are often higher in the understorey compared to the overstorey, the understorey’s influence on cycles of essential plant nutrients, including nitrogen and phosphorus, is often disproportionate to its relative biomass (Moore et al. 2007; Welch et al. 2007). Understorey plants are therefore expected to play an important role in preventing nutrient leaching. Especially in spring, when trees are still dormant and cannot sequester nutrients themselves, understorey plants can act as a temporary reservoir that prevents leaching of nutrients (Tessier and Raynal 2003). Knowledge on how nutrient and carbon cycling (i.e. the annual input of carbon and nutrients into the soil from dying plant material) by the understorey varies across overstorey types will be crucial to quantify potential trade-offs or synergies between overstorey and understorey functioning, which need to be accounted for when optimizing forest ecosystem functioning (Landuyt et al. 2019a).

First of all, the overstorey strongly controls understorey biomass and nutrient stocks via regulating light availability, being the limiting resource for understorey carbon gain during the leaf-on period of the overstorey. Light availability at the forest floor is mainly driven by (1) tree architectural attributes such as basal area, crown structure, and leaf area index, (2) leaf morphological attributes such as specific leaf area and leaf size and (3) leaf phenology (e.g. Palik et al. 1997; Comeau and Heineman 2003), all depending on tree species composition, age structure and density. While the first two determine light availability during the overstorey’s growing season, the latter determines the variability of light availability over the course of a year. Evergreen tree species, for example, give rise to more stable light conditions throughout the year (Hamada et al. 2016).

The overstorey can additionally influence the understorey via its imprint on the soil (Augusto et al. 2003; De Schrijver et al. 2012; Cools et al. 2014). Overstorey litter with a low C to N ratio can significantly speed up litter decomposition and can hence determine soil nutrient availability and growth conditions, such as soil pH, aluminum toxicity and thickness of the litter layer (e.g. Edmonds 1980; Zhang et al. 2008). Similar as with light, the presence of evergreen species in the overstorey will influence interannual litter and soil nutrient dynamics. Evergreens release their leaves (and hence nutrients) more gradually, in contrast with the concentrated release of nutrients by temperate deciduous tree species (Zhang et al. 2014). Moreover, evergreen tree species often have lower specific leaf area and lower leaf mass-based nitrogen content (Ishida et al. 2006), leading to differences in nitrogen and phosphorus cycling (Son and Gower 1991).

Next to these tree species identity effects, as discussed above, also diversity effects can play an important role in determining the structure and composition of the understorey. According to the environmental heterogeneity hypothesis, mixing tree species induces a patchy pattern of environmental conditions that reflect tree species identity effects (Yankelevich et al. 2006). This pattern can be used by a larger number of species with different requirements or niches (Ellenberg and Leuschner 2010), compared to more homogenous conditions found in monocultures. In other words, more understorey species may find optimal growth conditions in mixed forest stands (Vockenhuber et al. 2011), potentially resulting in higher understorey biomass compared to monocultures (Zhang et al. 2017). In addition, Gartner and Cardon (2004) showed that leaf litter mixtures, as produced by stands with a high tree species diversity, often decompose faster, which leads to mull-type soil conditions that may increase herb layer biomass and nutrient content (e.g. Landuyt et al. 2019b).

In summary, previous studies have already shown that a range of overstorey characteristics can drive the structure and composition of the understorey. However, studies focusing on understorey biomass and its nutrient content, i.e. its importance for carbon and nutrient cycling in forest ecosystems, are still scarce, and did not study the individual importance of these overstorey factors in a joint analysis, across a range of forest types. Here we report such an analysis based on the FunDivEUROPE Exploratory Platform, a European research platform that permits disentangling effects of tree species diversity and composition on forest ecosystem functioning across six contrasting forest types (Baeten et al. 2013). We specifically investigate the following hypotheses:

1. The understorey’s contribution to nutrient and carbon cycling in forest ecosystems is non-negligible, but largely depends on the forest type considered. Across forest types, general trends are expected to emerge:
2. The importance of the understorey is mainly driven by light availability, leading to a more important functional role of the understorey in more open forest stands.
3. In addition to light, also overstorey litter quality and species richness will have a positive influence on the understorey’s functional importance.

To investigate these hypotheses, we focus on the mass and nutrient content of plant material that dies off every year, being the fallen leaves for the overstorey and the non-lignified aboveground biomass for the understorey, assuming that these compartments are the main contributors to annual carbon and nutrient cycling in temperate forest.

## Methods

### Site information

**Fig. 1:**
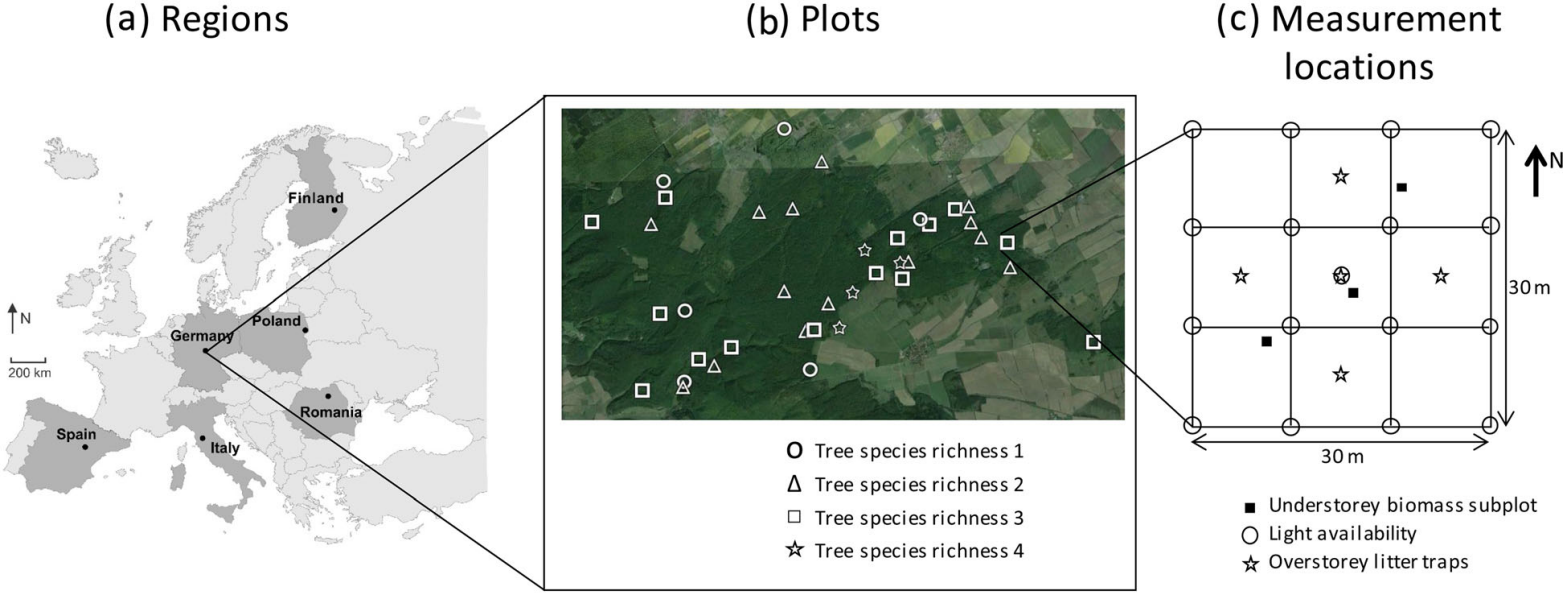
Overview of the FunDivEurope Exploratory Platform, with (**a**) an overview of the study regions indicated with black dots, (**b**) a zoom-in on the German Exploratory Platform, showing the location and tree species richness levels of all German plots (study area size 15 km × 10 km), and (**c**) a scheme of the plot lay-out, with the measurement locations (modiefied from Ampoorter et al. 2016)

The Exploratory Platform of the FunDivEUROPE project encompasses six major forest types across Europe, located along large soil and climatic gradients: boreal forest (Finland), hemiboreal, nemoral coniferous, mixed broadleaved-coniferous forest, hereafter referred to as “hemiboreal forest” (Poland), beech forest (Germany), mountainous beech forest (Romania), thermophilous deciduous forest (Italy) and Mediterranean mixed forest (Spain) (Fig. 1a). In each region, three to five target tree species were selected that are regionally common and/or of silvicultural importance. The total tree species pool consisted of coniferous species *Abies alba*, *Picea abies*, *Pinus nigra* and *Pinus sylvestris*, and broadleaved species *Acer pseudoplatanus*, *Betula pendula*/*pubescens*, *Carpinus betulus*, *Castanea sativa*, *Fagus sylvatica*, *Fraxinus excelsior*, *Ostrya carpinifolia*, *Quercus robur*/*petraea*, *Quercus cerris*, *Quercus faginea* and *Quercus ilex*. Depending on regional tree species richness, 28 to 43 plots were chosen per region (209 in total). In each region, plots cover a gradient in target tree species richness from monoculture stands to full mixtures of the target tree species. In mixed stands, tree species are intimately mixed, and contain different target tree species compositions per target tree species richness level (Fig. 1b). This set-up allowed separation of target tree species identity and diversity effects. Admixtures of non-target tree species were accepted as long as the summed basal area of the admixed species was below 5% of the total basal area. Plot size was 30 m × 30 m and each plot was subdivided in nine quadrats of 10 m × 10 m (Fig. 1c). All study forests are considered ancient forest, i.e. they have been continuously forested since the oldest available land-use map, with no signs of recent management. All forests are either in the mid to late stem exclusion, understorey reinitiation or old-growth development stage (i.e. excluding very young stands). For more information on the regions, plot selection criteria and plot-level information, see Baeten et al. (2013) and Jucker et al. (2014).

### Measurements of understorey biomass and nutrient concentrations

In the southwestern, central and northeastern quadrat of every plot, one subplot of 0.5 m × 0.5 m was delineated using a wooden frame, at a position where the understorey composition/cover was largely representative for the quadrat (Fig. 1c). In these subplots, we clipped the understorey aboveground biomass (< 1.3 m) in 2012 (Germany, Italy and Poland in May, Spain in June, Finland and Romania in August). As it is mainly the non-lignified biomass that contributes to carbon and nutrient cycling, biomass samples were sorted into ‘lignified’ (lignified seedlings, lignified perennial plants) and ‘non-lignified’ biomass fractions (annual plants, young non-lignified seedlings, young non-lignified perennial plants) that were dried for 48 h at 70 °C and weighed (*dry weight*). The non-lignified biomass fraction was grounded and analysed for C, N and P, as it is mainly this biomass compartment that contributes to yearly carbon and nutrient cycling in forests. We measured carbon (C) and nitrogen (N) concentrations by high temperature combustion at 1150 °C using an elemental analyzer (Vario MACRO cube, Elementar, Germany). Phosphorus (P) concentration was measured colorimetrically according to the malachite green procedure (Lajtha et al. 1999) after digesting 100 mg of the sample with 0.4 mL HClO_4_ (65%) and 2 mL HNO_3_ (70%) in Teflon bombs for 4 h at 140 °C. Subplot-level nutrient stocks were calculated multiplying the dry weight of the understorey non-lignified biomass by the nutrient concentrations, and represent annual nutrient fluxes towards the soil. Plot-level values were obtained by averaging biomass, nutrient concentration and stock estimates of the three subplots and rescaling them from 0.25 to 1 m^2^. Note, however, that these estimates of the understorey’s contribution to carbon and nutrient cycling, do not account for resorption of nutrients prior to leaf senescence and are therefore expected to slightly overestimate the contribution of the understorey to these cycles.

### Measurements of overstorey leaf litterfall and nutrient concentrations

Overstorey litterfall was collected from autumn 2012 till autumn 2013 in five litter traps that were placed systematically within each plot (Fig. 1c). A litter trap consisted of a bag of approximately 0.5 m depth and an opening area of 0.5 m^2^, without soil contact to avoid decomposition processes by soil microorganisms. Litterfall was collected at least once before snowfall, once in spring as soon as snow cover disappeared and plots were accessible, and then ideally on a monthly basis during the rest of the year, to avoid pre-collection decomposition. After each collection period, samples of the five litter traps per plot were pooled, dried for 48 h at 38 °C and sorted into different fractions (foliar litter target tree species, foliar litter other tree species, woody litter, reproductive litter and a rest fraction). Chemical analyses were performed on the foliar litter of the target tree species only, for each species separately. For Germany and Italy, one sampling period in autumn was selected to examine the chemical constitution of the leaf litter. Concerning Finland, Poland, Romania and Spain, for deciduous tree species chemical analysis was done on samples collected in autumn, while for evergreen tree species chemical analysis was performed on samples collected during spring, summer or autumn, depending on peak litterfall periods of the respective species. Deviations from this general rule were sometimes necessary due to practical reasons (e.g. first litter sample too small for chemical analyses). The target tree species’ foliar litter subsamples were ground using a ball mill and chemically analysed for C and N concentration by Near Infra-Red Spectroscopy, as described by Niederberger et al. (2015). In order to calibrate the Near Infra-Red Spectroscopy spectra and validate the calibration for the determination of N concentration, a subset of samples was analysed with a flash CHN Elemental Analyser (Flash EA1112 Series, ThermoFinnigan, Milan, Italy). The procedure of spectra calibration and Near Infra-Red Spectroscopy application is described by Pollastrini et al. (2016). The other litterfall fractions generally represented a minority of the total overstorey litterfall, although this proportion reached 51% in the hemiboreal forest in Poland (for more information see Fig. S1). Plot-level C and N concentrations in overstorey foliar litterfall were calculated as the weighted mean of the C and N concentrations assessed for each target tree species, using the total dry weight of the foliar litter of each target tree species collected throughout the year as a weighting factor. The nutrient stocks in the foliar litterfall of all target tree species were calculated by multiplying foliar nutrient concentration by foliar dry weight for each target species and by summing these across all target tree species. Methodological differences in analyses of understorey and overstorey deciduous biomass were due to the fact that the work was done by different teams at different points in time.

### Measurements of light availability

Light availability was measured in all regions except for Italy. At every quadrat corner and in the plot center (17 points in total), we took a hemispherical photograph using a horizontally-levelled digital camera (CoolPix 995, Nikon, Tokio, Japan), mounted on a tripod and aimed at the zenith, using a fish-eye lens of 180° field of view (FCE8, Nikon) (Valladares and Guzmán 2006) (Fig. 1c). Photographs were taken at sunrise and sunset, minimising variation due to exposure and contrast. Moreover, in each forest, they were taken when trees had fully expanded leaves, i.e. Spain in June, Germany in July, Romania and Finland in August 2013 and finally, Poland in August 2014. We analysed all photographs using the software Hemiview v.2.1 (Delta-T Devices Ltd., Burwell, UK) that determines a Direct and Indirect Site Factor as proxies for the direct and indirect light availability, respectively (Rich 1990). A Global Site Factor was then calculated as 0.9 × (Direct Site Factor) + 0.1 × (Indirect Site Factor), representing the total light availability (Valladares and Guzmán 2006). Plot-level light availability was calculated as the average of the 17 values obtained from the 17 pictures within a plot.

### Data analysis

In a first exploratory step, plot-level weights of overstorey leaf litterfall and understorey non-lignified biomass were plotted to visualize the understorey’s importance for carbon cycling. We additionally explored the relative contribution of the understorey to plot-level nutrient cycling, by comparing P and N concentrations and stocks in overstorey leaf litterfall with those measured in the non-lignified fraction of understorey biomass. As the selected forest types differ considerably in terms of soil type, climatic conditions and species composition, we visualized the importance of the understorey for carbon and nutrient cycling for each forest type. Analysis of variance was used to assess significant differences among forest types, separately for overstorey and understorey estimates.

In a second step, we ran linear models to analyse the effect of light availability, target tree species richness, overstorey litter quality and the proportion of evergreen tree species (all included as main effects) on understorey (non-lignified) biomass and N and P concentrations. Overstorey litter quality was quantified as the C/N ratio, using the weighted average plot-level C and N concentrations of the target tree species foliar litter. The proportion of evergreen tree species was quantified based on their contribution to the total plot-level basal area, while light availability was quantified in terms of Global Site Factor. As we assume that species richness effects are not necessarily proportional to the number of species, species richness was deliberately included as a factor instead of a numeric predictor. To account for potential forest type specific effect sizes, we included forest type as an additional main effect and allowed it to interact with the other predictors, while among predictor interactions (e.g. light availability × litter quality) were not included.

Redundant predictors were removed from the full model by retaining the model with the highest AIC after testing all possible removals of predictors and interactions (using the dredge function, R package MuMIn (Bartón 2013)). We followed a similar procedure to construct models for a range of other response variables, including understorey nutrient stocks (N and P) and the relative contribution of the understorey, in terms of biomass (non-lignified understorey biomass/biomass of leaves in overstorey litterfall) and nutrient stocks (understorey N stock/overstorey N stock) (for results, see Fig. S4). Prior to the modelling step, all variables were scaled, checked for outliers and tested for potential correlations. Spearman correlation coefficients were < 0.6 (ranging between − 0.33 and 0.58, Fig. S2), so all predictor variables were retained in the models. All analyses were performed in R 3.4.3 (R Core Team 2020).

## Results

**Fig. 2:**
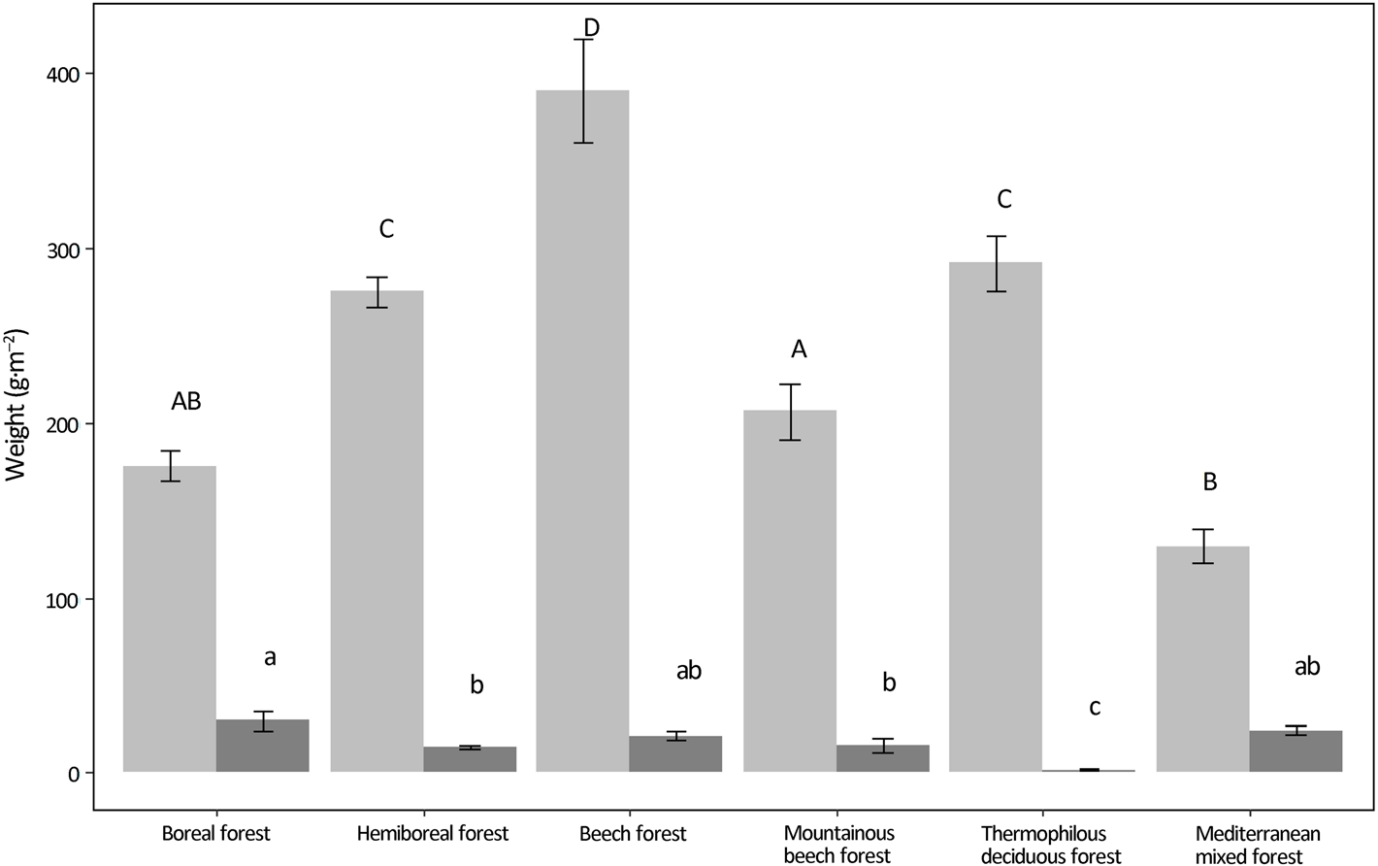
Amount of overstorey foliar litterfall (target tree species) and understorey non-lignified biomass per area unit across the studied forest types. Bars represent the mean ± standard error of the mean. Letters above the bars denote significant differences across forest types, for overstorey (uppercase) and understorey (lowercase) variables

In all forest types, total annual leaf litter production (of the target tree species) clearly exceeded the non-lignified aboveground understorey biomass (Fig. 2). The relative importance of both biomass pools, however, depended on the forest type. The contribution of the understorey was the lowest for the thermophilous deciduous forests in Italy, and the highest for Mediterranean mixed forests in Spain. Changes in the understorey’s relative contribution mainly depended upon litter production in the overstorey, with lower overstorey litter production leading to a higher contribution of the understorey (Fig. 2).

**Fig. 3:**
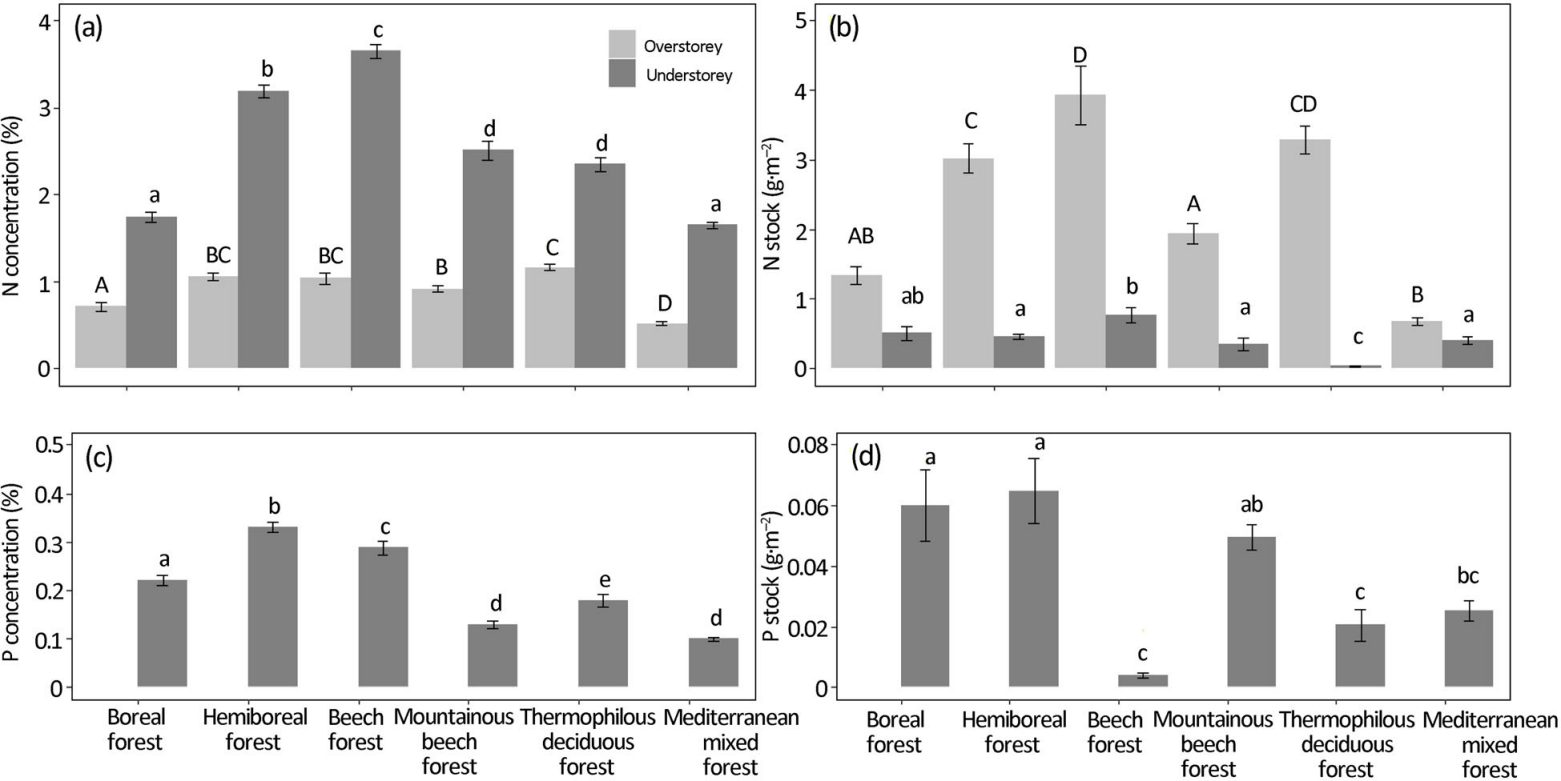
Nutrient concentrations and stocks in overstorey foliar litterfall and understorey non-lignified biomass: (**a**) nitrogen concentration, (**b**) nitrogen stocks, (**c**) phosphorous concentration and (**d**) phosphorous stocks. Bars represent the mean ± standard error of the mean. Phosphorous concentration and stock were not determined for overstorey foliar litterfall. Letters above the bars denote significant differences across forest types, for overstorey (uppercase) and understorey (lowercase) variables

In terms of nutrient stocks, the relative contribution of the understorey became more important due to a higher understorey nutrient concentration (Fig. 3). Nitrogen concentrations in non-lignified understorey biomass (ranging between 1.65% and 3.65%) were, on average, three times higher than concentrations measured in fallen overstorey leaves (ranging between 0.52% and 1.16%) (Fig. 3a). Measured nutrient concentrations, however, differed among forest types. For most forest types, the relative contribution of the understorey’s non-lignified biomass to the total N stock in non-lignified understorey biomass and overstorey foliar litterfall was relatively low, ranging between 1% and 16%. Except for the boreal and the Mediterranean mixed forests, the understorey’s contribution to this N stock reached 28% and 37%, respectively (Fig. 3b). Similar to N concentrations, also P concentrations in the understorey’s non-lignified biomass differed among forest types (Fig. 3c and d).

**Fig. 4:**
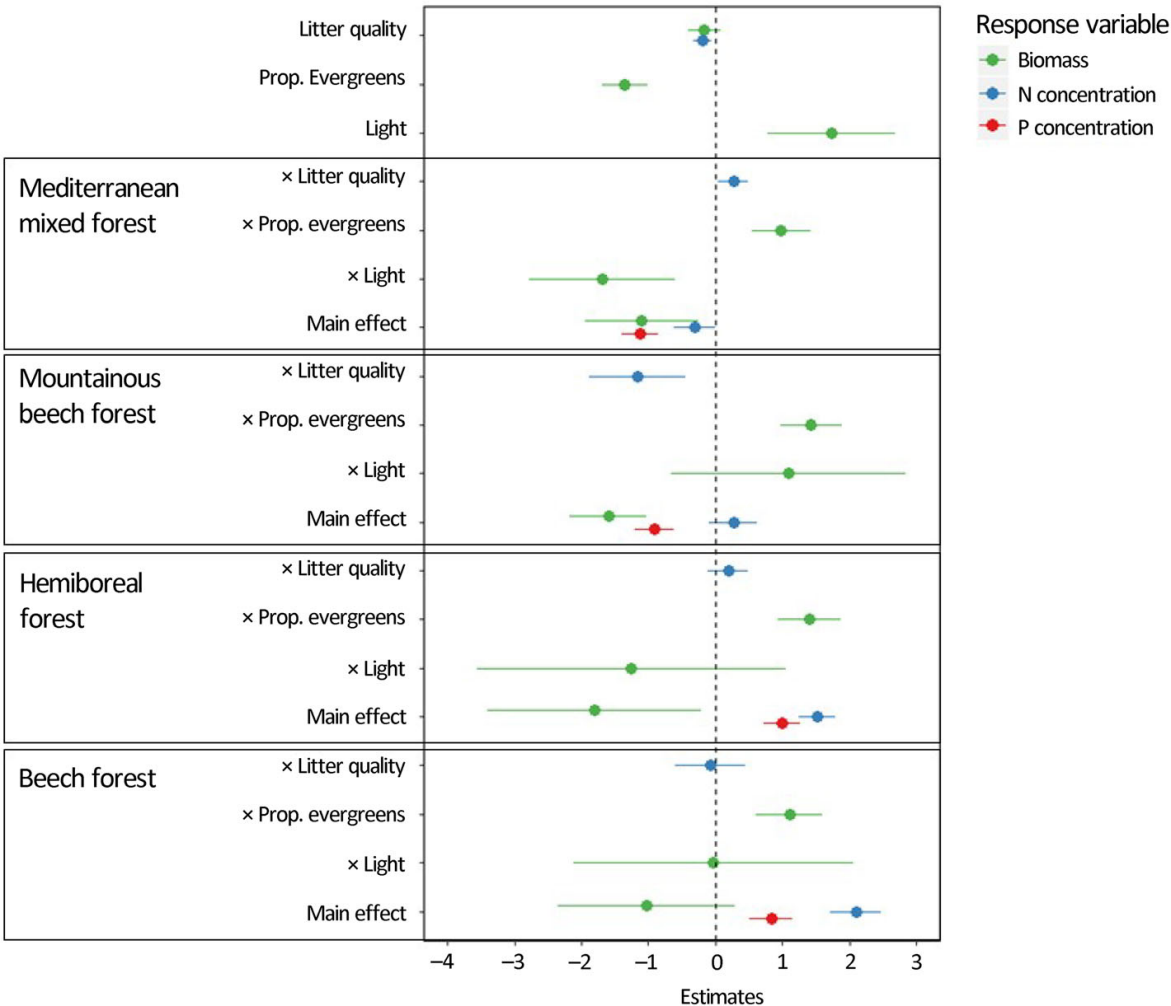
Estimated model coefficients for linear models predicting understorey non-lignified biomass (*R*^2^ = 0.60), understorey phosphorus (P) concentration (*R*^2^ = 0.75) and understorey nitrogen (N) concentration (*R*^2^ = 0.83). Missing coefficients for some predictors indicate that these terms were not retained in the final models. All forest type coefficients should be interpreted relative to the reference, being the boreal forests in Finland. Error bars indicate 95% confidence intervals. When these include zero, effects can be considered non-significant

**Fig. 5:**
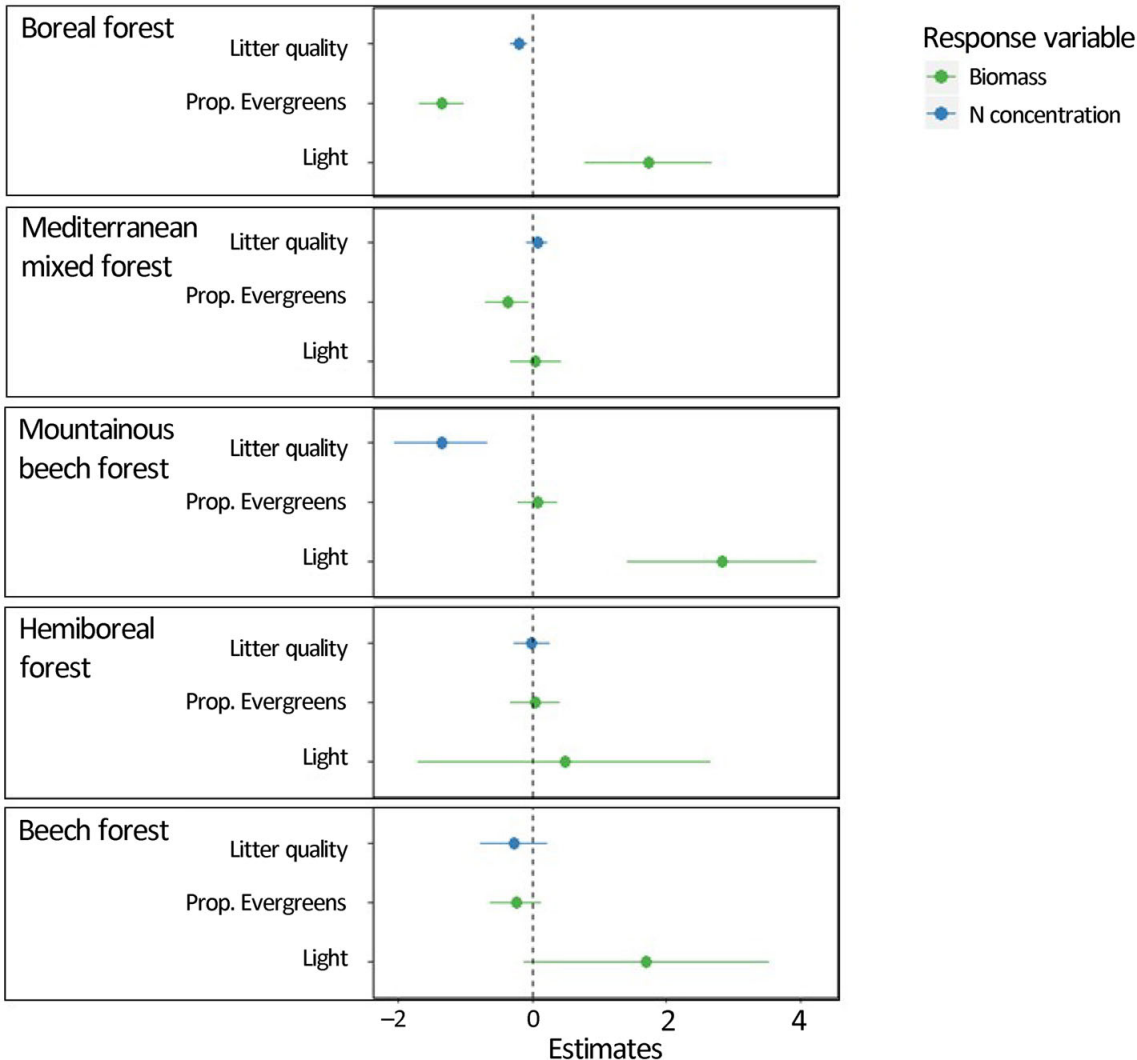
Overview of all retained interaction terms and their influence on the total effect sizes of the retained predictor variables in the different forest types. Effect sizes were calculated by summing the estimated coefficients for the main and interactive effects, depicted in Fig. 4. Standard errors were calculated based on the models’ covariance matrices. Error bars denote 95% confidence intervals

Statistical modelling revealed the main drivers of understorey biomass and nutrient concentrations in the investigated forests (Fig. 4). Forest type was an important predictor for all considered response variables, namely understorey biomass, P concentration and N concentration. Forest type came out as a main effect, but often also interacted with other drivers. While the main effect of the proportion of evergreen species in the overstorey on understorey biomass was negative (Fig. 4), significant interactions with forest type indicated different effects depending on the forest type, with strong negative effects in boreal forests, and weaker or no effects in the other forest types (Fig. 5). Also the effects of overstorey litter quality, expressed in terms of overstorey leaf litter C to N ratio, on understorey N concentrations differed depending on the forest type, with strong positive effects in the mountainous beech forests in Romania only, where a high overstorey litter quality (low C/N) led to a higher N concentration in the understorey (Fig. 5). Finally, also the effect of light on understorey biomass, which was in general positive as expected, differed depending on the forest type. Especially in Mediterranean forests, the interaction term compensated the main effect of light, leading to no net positive effect of light availability on understorey biomass (Fig. 5). Considering all predictors, our model results additionally suggest that forest types primarily drive understorey nutrient concentrations, while local forest characteristics (proportion of evergreen species and light availability) drive understorey biomass. Surprisingly, overstorey species richness was not retained as a predictor in any of our models, suggesting that direct effects of overstorey species richness on understorey biomass and nutrient concentrations are absent.

## Discussion

It is generally accepted that the overstorey is the most important contributor to litter production and nutrient cycling. We here tried to quantify this contribution by comparing the biomass and nutrient content of the non-lignified biomass of the understorey to that of leaf litter produced by the overstorey. Our results confirmed that overstorey litterfall plays a dominant role in carbon and nutrient cycling, but also showed that the share of the understorey should not be neglected. We also found substantial differences across forest types. In temperate forests, the overstorey was by far the major contributor, while in boreal and Mediterranean mixed forests, the contribution of the understorey to carbon and nutrient cycling was more important. Across all forest types, light availability and the proportion of evergreen species were the main drivers of understorey biomass, while forest type was the dominant driver for nutrient concentrations in the understorey. Contrary to our expectations, overstorey species richness did not affect understorey biomass, nor its nutrient content.

### The importance of the understorey for litter production and nutrient cycling

Annual litter production of the overstorey within the FunDivEUROPE Exploratory Platform (around 3.6 t·ha^− 1^·year^− 1^, see also Fig. S1) had the same order of magnitude as found in the literature review by Sayer (2006). Also the total aboveground understorey biomass found in our plots (around 0.4 t·ha^− 1^) matched the figures shown by Mölder et al. (2008) and Axmanová et al. (2012), indicating a relatively low contribution of the understorey to annual litter production in forests. This contribution, however, varied among our studied forest types. Forest types occurring in mild climates had closed canopies, produced a somewhat higher amount of overstorey litterfall and were characterized by a low biomass understorey. In contrast, forest types occurring in harsher conditions, i.e. drier (Mediterranean mixed forests in Spain) or colder (boreal forests in Finland), had a rather open canopy (thin crown and/or low basal area), produced a lower amount of overstorey litterfall, and were characterized by a lush understorey layer. Although our data show that the understorey becomes more important in forest types where the overstorey is less productive, our results also show that the understorey cannot fully compensate for the loss of overstorey litter production in more open forest types, resulting in a lower total litter production in more open forests.

We found that the non-lignified biomass of the understorey had a higher nutrient content compared to overstorey leaf litter, again confirming previous studies (e.g. Aubert et al. 2005; Moore et al. 2007; Golay et al. 2016). Although the understorey’s contribution to nutrient cycling exceeds its contribution to litter production, it still remains under the contribution of the overstorey in all forest types.

### The main drivers of understorey litter production and nutrient cycling

Light availability was an important predictor for the amount of non-lignified understorey biomass, confirming previous studies that pointed at light as the main limiting factor for biomass production in forest understoreys (Axmanová et al. 2011, 2012). However, the effect of light was not pervasive, as in the Mediterranean mixed forests in Spain, an increase of light availability did not imply higher understorey biomass. An explanation can be that in dry climatic conditions an increase of light availability normally induces water stress due to increased evapotranspiration rates (Valladares et al. 2016), leading to a lower understorey productivity. This is supported by Bastias et al. (2019) who found the lowest values for tree recruitment in the understorey of these two same forest types (Mediterranean and boreal forests) situated at the extreme of the latitudinal gradient.

Contrary to our third hypothesis, the quality of overstorey litter did not influence understorey biomass or its contribution to litter production. This again confirms that light availability is the main limiting resource for understorey productivity (Gilliam and Turrill 1993; Axmanová et al. 2011; Smolko and Veselovská 2018; Landuyt et al. 2019b). We did find a general positive relationship between overstorey litter quality (with a low C/N indicating a high litter quality) and understorey N concentration. This result suggests that the overstorey can enhance nutrient cycling, not only by producing high quality litter, but also by stimulating the nutrient cycling capacity of the understorey when producing high quality litter. This relationship was the strongest in mountainous beech forests. Mechanisms behind these relationships are probably related to soil nutrient availability, as found by Landuyt et al. (2019b). High quality overstorey litter can increase soil nutrient availability, which promotes nutrient uptake by understorey plants. Limited availability of light might be the reason that this enhanced uptake of nutrients does not lead to an enhanced productivity of the understorey.

High proportion of evergreen species in the overstorey affected understorey biomass negatively. However, significant interactions with forest type compensated this main effect for all but the reference forest type, being the boreal forests in Finland (Fig. 5). Hence, it is mainly in these boreal forests that an increasing proportion of evergreen species decreased understorey biomass. As all evergreen species in these forests were coniferous, this effect can be considered a conifer effect. Previous studies already pointed at lower light and soil nutrient availability in coniferous forests, compared to broadleaved forests, negatively affecting understorey biomass production, nutrient concentrations and stocks (e.g. Messier et al. 1998; Koorem et al. 2011). In the other forest types, evergreen species were either present at a lower density (Fig. S3) or represented by broad-leaved species (e.g. *Quercus ilex* in Mediterranean forests). Differences between broad-leaved and needle-leaved species in terms of leaf chemistry and their imprints on the soil can potentially explain the weaker relationships found in these forests (Takahashi 1997).

We found no significant relation between target tree species richness and understorey biomass. With these results we confirm previous studies that did not find any affect (e.g. Zhang et al. 2016), but also contradict others that either found positive or negative effects (e.g. Cavard et al. 2011; Zhang et al. 2016). These contrasting findings indicate that this relationship is either context-dependent or hard to detect due to potential interactions or confounding with other predictors. As we did not find a relation in any of the forest types investigated, we did not found evidence for this context-dependency. Another potential reason for lacking overstorey diversity effects might be the presence of counteracting effects, as suggested by Zhang et al. (2016). Mixing tree species may lead to higher overstorey biomass production due to greater use of available resources (Forrester and Bauhus 2016). Consequently, this higher resource use by overstorey trees in mixed forest stands may reduce the amount of resources still available for the understorey, neutralising the positive mixing effects as discussed in the introduction. Biomass production in the understorey may thus appear unaltered or even reduced compared to the corresponding monocultures (Zhang and Chen 2015).

### Main limitations of the study and potential implications

A first limitation of our study is that only one field campaign was conducted per region. Hence, we estimated peak understorey biomass, but missed the biomass of understorey species that appear earlier or later in the growing season. As these species also contribute to litter production and nutrient cycling, our findings likely underestimated the importance of the understorey, especially in forests with a rich vernal flora (such as *Allium ursinum* in beech forests on calcareous soils). Multiple field visits throughout the year could be an easy way to deal with this issue in future studies. Second, nutrient concentrations for both the understorey and the overstorey were measured on a fraction of their total biomass only, being the non-lignified aboveground biomass for the understorey and leaf litterfall only for the overstorey. These fractions, however, are not the only ones that contribute to annual litter production and nutrient cycling. Consequently, our estimated nutrient stocks slightly underestimated the functional importance of the overstorey and the understorey for nutrient cycling. Nevertheless, leaf litter was the dominant fraction of overstorey litterfall across all forest types (Fig. S1), and non-lignified understorey biomass can be considered the most important component of understorey litter production. These fractions and their nutrient concentrations can therefore be considered representative measures to assess the functional role of the overstorey and the understorey in terms of litter production and nutrient cycling. Finally, we collected foliar litter that was shed by the trees, after nutrient resorption. In contrast, we clipped the aboveground understorey biomass when it was expected to be at maximum coverage. During this period, understorey nutrient concentrations are at their maximum as resorption of nutrients in the (lignified and non-lignified) perennial plants generally takes place later in the growing season. Due to the mismatch between clipping (in spring) and nutrient resorption (in autumn) for the understorey, we probably overestimated N and P concentrations and stocks in the understorey. Future research, studying the extent and timing of nutrient resorption in the understorey could shed more light on this issue.

## Conclusions

Our results showed that the understorey’s contribution to litter production and nutrient cycling in European forests should not be neglected, with a contribution ranging between 0.5% and 16% for litter production, and between 1% and 37% for N cycling. These values mainly depended on the forest type considered and were, across forest types, largely determined by the availability of light for the understorey. This finding, that the understorey’s functional role becomes more important when light availability increases, points at a trade-off between overstorey and understorey functioning, that needs to be accounted for when optimizing forest ecosystem functioning. On the other hand, the lower sensitivity of the understorey to the other drivers, including tree species richness, tree litter quality and the proportion of evergreen species in the overstorey, indicate that overstorey functioning can still be optimized (e.g. by manipulating these overstorey attributes within the investigated ranges) without constraining the functionality of the understorey layer.

## Supplementary information

**Additional file 1:Figure S1.** Plot-level weight of the different overstorey litter fractions and understorey biomass fractions in the different forest types/regions. **Figure S2.** Correlogram showing correlations between predictor variables light availability, target tree species richness, overstorey foliar litter C to N ratio and the proportion of evergreen tree species. **Figure S3.** Distribution of light availability, expressed as Global Site Factor, overstorey litter quality, expressed as carbon to nitrogen ratio, and proportion of evergreen tree species between and within regions. **Figure S4.** Estimated model coefficients for linear models predicting understorey phosphorus stock, understorey nitrogen stock and the understorey’s relative contribution to litter production and nitrogen fluxes.

## Data Availability

The datasets used and/or analysed during the current study are available from the corresponding author on reasonable request.
