# Supplementary material for: Importance of overstorey attributes for understorey litter production and nutrient cycling in European forests
Source: For Ecosyst. 2020 Jul 12;7(1):45. doi: 10.1186/s40663-020-00256-x (PMC7357776; doi:10.1186/s40663-020-00256-x)
Supplement: Supplementary file 1 — Additional file 1:Figure S1. Plot-level weight of the different overstorey litter fractions and understorey biomass fractions in the different forest types/regions. Figure S2. Correlogram showing correlations between predictor variables light availability, target tree species richness, overstorey foliar litter C to N ratio and the proportion of evergreen tree species. Figure S3. Distribution of light availability, expressed as Global Site Factor, overstorey litter quality, expressed as carbon to nitrogen ratio, and proportion of evergreen tree species between and within regions. Figure S4. Estimated model coefficients for linear models predicting understorey phosphorus stock, understorey nitrogen stock and the understorey’s relative contribution to litter production and nitrogen fluxes. [file 40663_2020_256_MOESM1_ESM.docx]

**Importance of overstorey attributes for understorey litter production and nutrient cycling in European forests**

**Supporting Information**

**Figure S1** Plot-level weight of the different overstorey litter fractions and understorey biomass fractions. Bars represent the mean ± standard error of the mean. Different panels represent different regions, each containing a different forest type: boreal forest (Finland), hemiboreal, nemoral coniferous, mixed broadleaved-coniferous forest (Poland), beech forest (Germany), mountainous beech forest (Romania), thermophilous deciduous forest (Italy) and Mediterranean mixed forest (Spain). While foliar litter of the target tree species was dried at 38°C, all other litter fractions were dried at 65°C to obtain constant weight. To enable comparing both litter pools, we dried a subsample of each target tree species until 65°C and assessed a dry weight conversion factor that was multiplied to weights obtained at 38°C. All data in the figure refer to dry weights at 65°C.

**
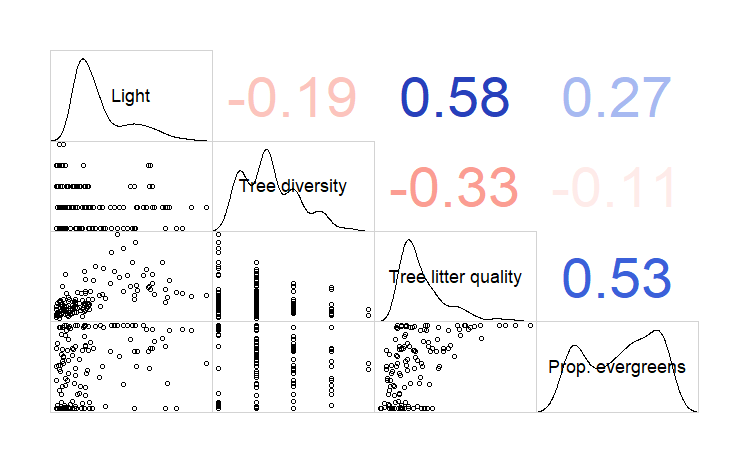
**

**Figure S2**  Correlogram showing correlations between predictor variables light availability (Light), target tree species richness (Tree diversity), overstorey foliar litter C to N ratio (litter quality) and the proportion of evergreen tree species (Prop. evergreens). The upper panels show Spearman correlation coefficients, the diagonal represents density plots and the lower panels show scatter plots.

**Figure S3** Distribution of (A) light availability, expressed as Global Site Factor, (B) overstorey litter quality, expressed as carbon (C) to nitrogen (N) ratio, and (C) proportion of evergreen tree species between and within each region.


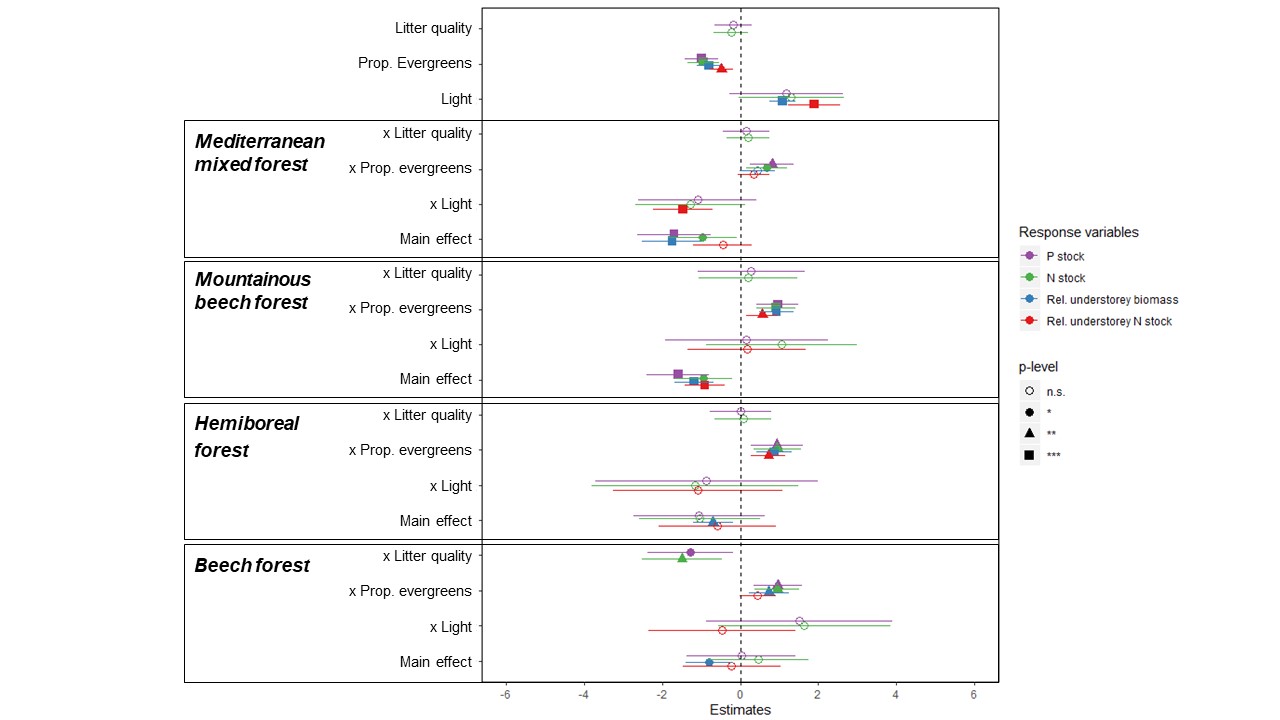


**Figure S4** Estimated model coefficients for linear models predicting understorey phosphorus stock, understorey nitrogen stock and the understorey’s relative contribution to litter production and nitrogen fluxes. Missing coefficients for some predictors indicate that these terms were not retained in the final models. All forest type coefficients should be interpreted relative to the reference, being the boreal forests in Finland. *, ** and *** correspond to *p*-values that are lower than 0.05, 0.01 and 0.001, respectively.
